# Intravascular Papillary Endothelial Hyperplasia: Diagnostic Sequence and Literature Review of an Orofacial Lesion

**DOI:** 10.1155/2014/934593

**Published:** 2014-05-07

**Authors:** Mahima V. Guledgud, Karthikeya Patil, Degala Saikrishna, Abhishek Madhavan, Tejesh Yelamali

**Affiliations:** ^1^Department of Oral Medicine & Radiology, JSS Dental College and Hospital, JSS University, Mysore 570015, India; ^2^Department of Oral & Maxillofacial Surgery, JSS Dental College and Hospital, JSS University, Mysore 570015, India

## Abstract

Intravascular papillary endothelial hyperplasia or Masson's tumor is a rare reactive disease of vascular origin characterized by exuberant proliferation of endothelial cells notably occurring within blood vessels of head, neck, and extremities. The importance of this entity is its ability to mimic a variety of diseases both benign and malignant in the orofacial region. Here, we present a case of Masson's tumor within the masseter muscle in a 40-year-old female with emphasis on the sequential investigative procedures performed to diagnose this entity.

## 1. Introduction

Vascular tumors in the oral region have been traditionally described as hamartomas or malformations rather than as true neoplasms. Stout stated that a vascular tumor, in contrast to a hamartoma, contained more endothelial cells than was necessary to line the lumina. Clinically, vascular lesions have a tumor-like appearance, due to endothelial proliferation in vessels and enlargement of vessels with secondary reactive change [1]. Intravascular papillary endothelial hyperplasia (IPEH) is a benign, nonspecific, vascular lesion consisting of reactive proliferation of the endothelial cells that arise in organizing thrombus [2]. It comprises approximately 2% of the vascular tumors of the skin and subcutaneous tissue [3].

## 2. Case Report

A 40-year-old female patient reported with a complaint of pain and increased swelling on the right side of the face of 1-week duration. The swelling was first noticed by the patient on the right cheek region 4 years back and was not associated with any trauma or chronic irritation. The swelling had gradually increased in size and was asymptomatic until a week before reporting to us during which the patient claimed that the swelling exhibited rapid increase in size and was associated with continuous moderate pain which aggravated on manipulation. No meal time variation in size of swelling was noted and patient did not give history of dryness of mouth. The patient's medical history was otherwise noncontributory.

**Figure 1:**
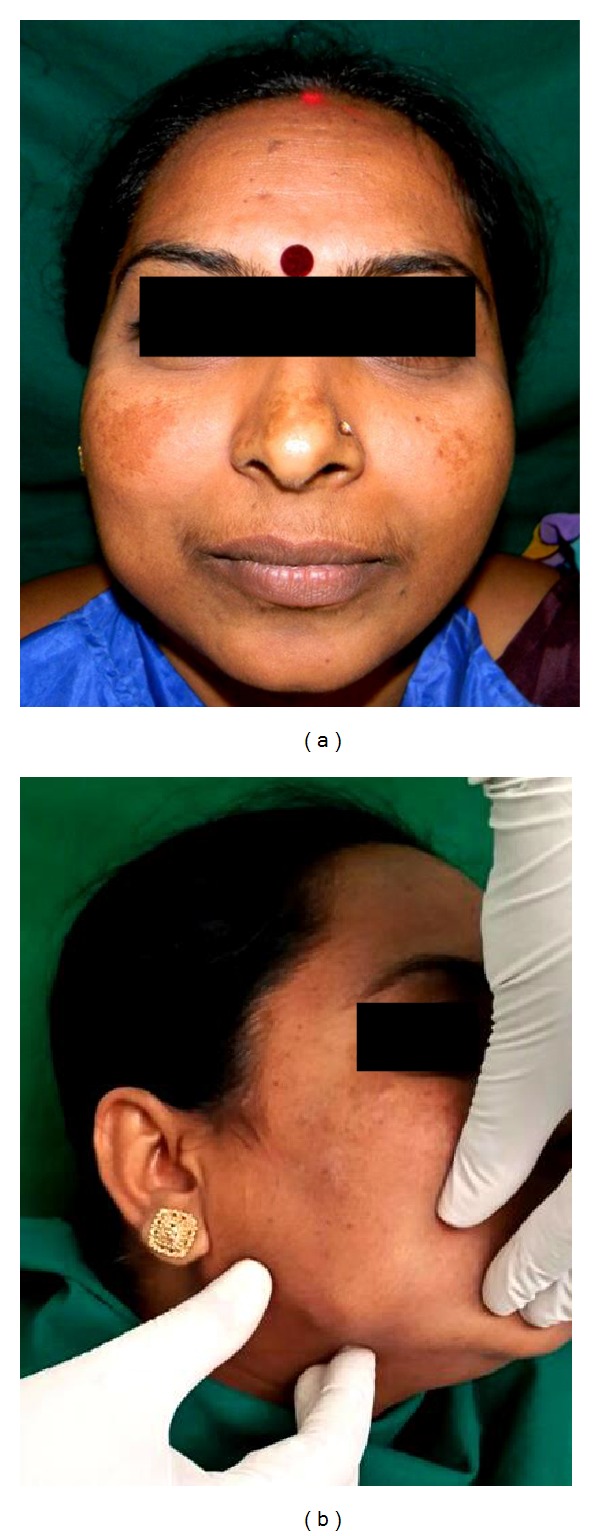
Extraoral frontal and right lateral view depicting the diffuse swelling on the right side of the face.

**Figure 2:**
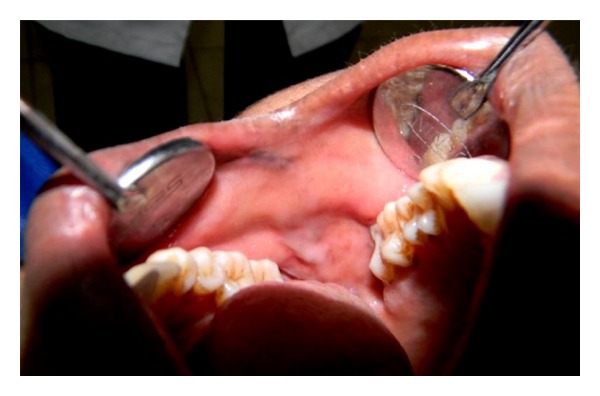
Intraoral right buccal mucosa exhibiting fullness.

Clinical examination revealed a solitary diffuse extraoral swelling on the right cheek, measuring about 2 × 2 cm. The skin overlying the swelling was normal with no secondary changes. On palpation, the swelling was tender, with no local rise in temperature, soft to firm in consistency, and lobulated with a smaller hard nodule palpable at the inferior portion of the swelling (Figure 1). The swelling was nonfluctuant, nonreducible, noncompressible, and nonpulsatile. The swelling was not fixed to any underlying or overlying structures and was freely mobile. There were no signs of altered sensations in the area involved. Intraorally a mild fullness of the right buccal mucosa was noted with no secondary changes with similar palpatory findings as performed extraorally (Figure 2).

**Figure 3:**
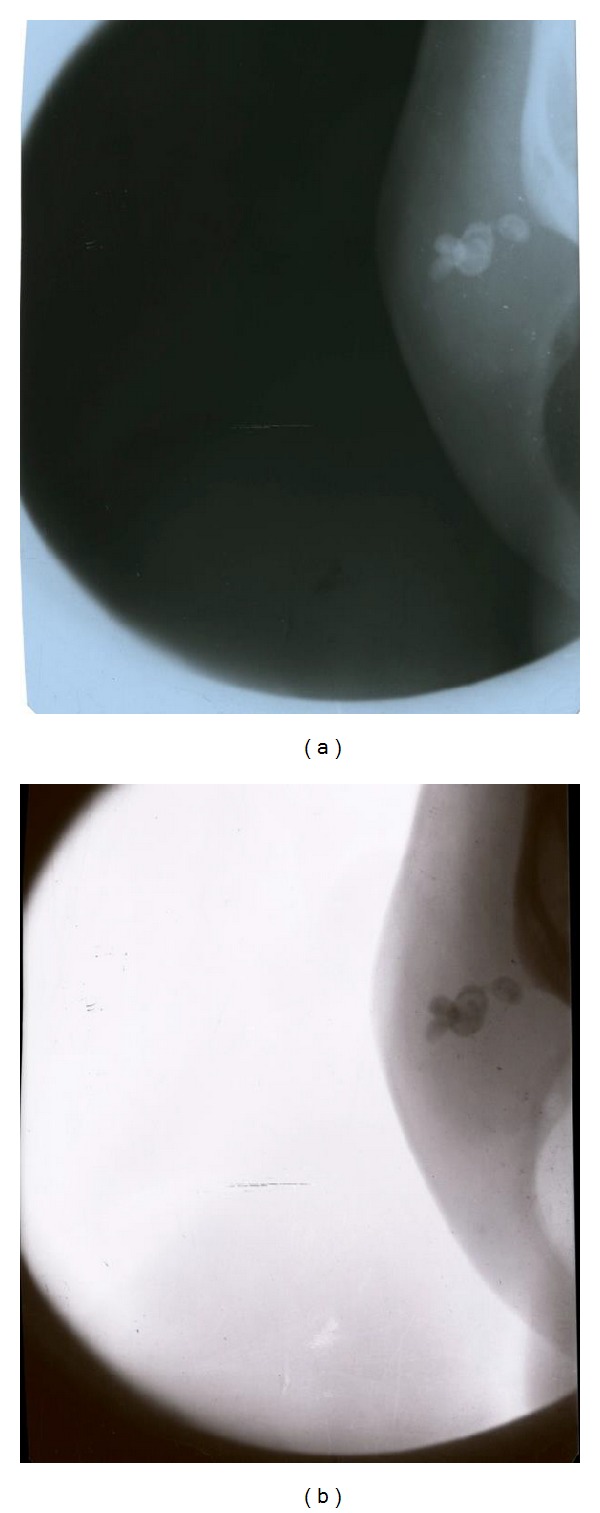
Puffed cheek radiograph and its inverted image depicting the calcifications noted within the lesion.

**Figure 4:**
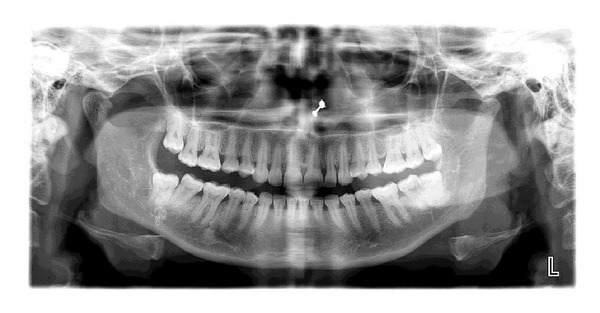
Panoramic radiograph revealing calcifications superimposed over the partially erupted lower right third molar.

**Figure 5:**
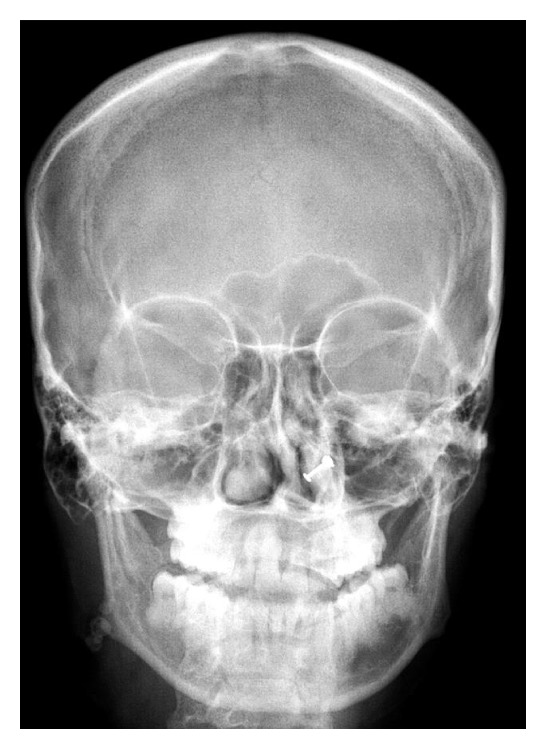
PA skull view showing the calcific structures in the right cheek region.

Soft tissue radiograph of the lesion using puffed cheek technique revealed 3 well-defined radiopaque concentric calcifications which were also visible on posteroanterior skull view and on the panoramic radiograph they were superimposed over the right mandibular third molar region (Figures 3, 4, and 5).

**Figure 6:**
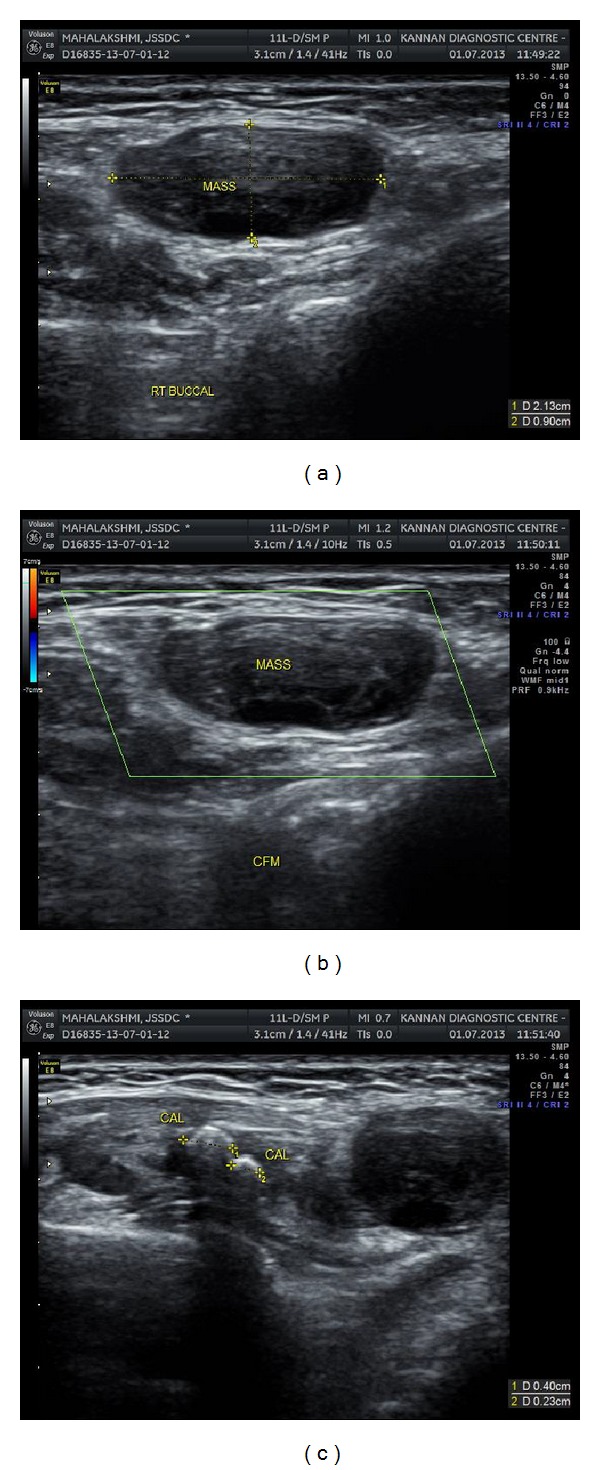
(a) Ultrasonography revealed a well-defined cystic mass within the masseter muscle. (b) Color doppler sonography indicated the mass to be avascular. (c) Ultrasonographic appearance of the calcific masses.

Ultrasonographic examination of the lesion revealed a fairly well-defined hypoechoic mass in the right cheek devoid of vascularity. Three foci of calcifications were noted within the right masseter muscle. The right parotid gland was normal in size and architecture (Figures 6(a), 6(b), and 6(c)).

**Figure 7:**
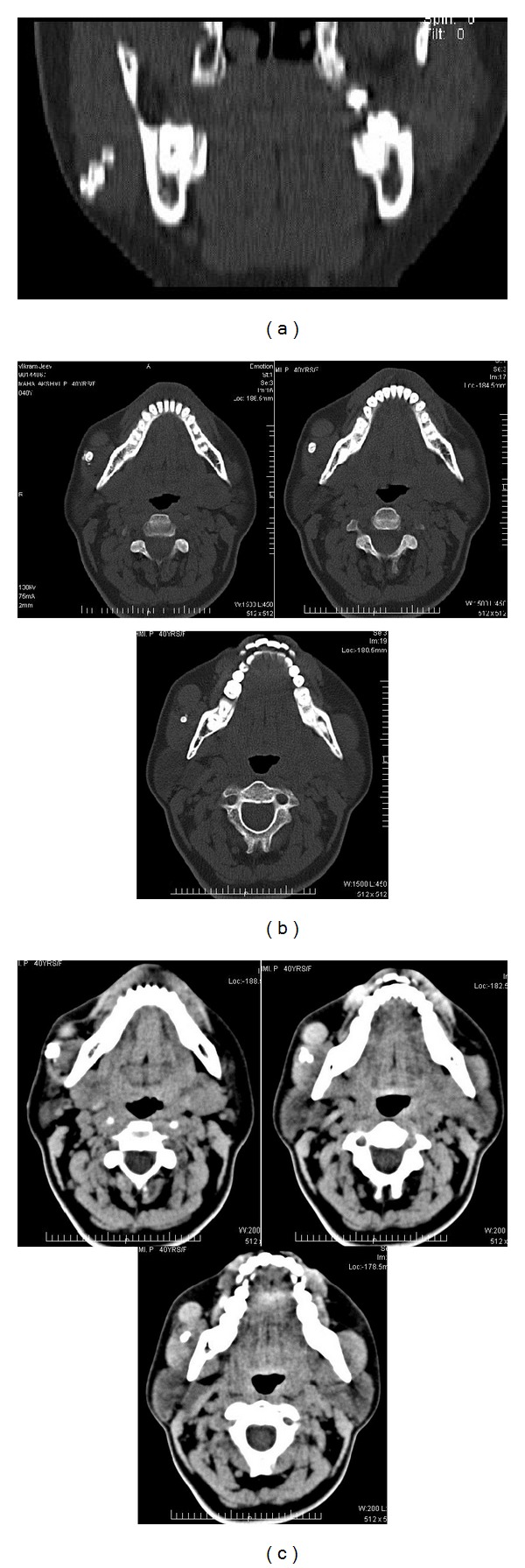
(a) Positions of the calcific structures noted in coronal CT section. (b) Bone window axial CT image shows position of the calcific structures at various depths. (c) Soft tissue window axial CT image clearly showing the boundaries of the mass.

CT revealed a well-marginated round to oval iso to hyperdense lesion seen anterior to the right masseter muscle measuring 1.5 × 1.3 cm with well-defined planes and attenuation values of around 100 HU. The lesion was seen to have continuation with anterior aspect of the masseter muscle which also exhibited irregular hyperdense calcific foci (Figures 7(a), 7(b), and 7(c)).

**Figure 8:**
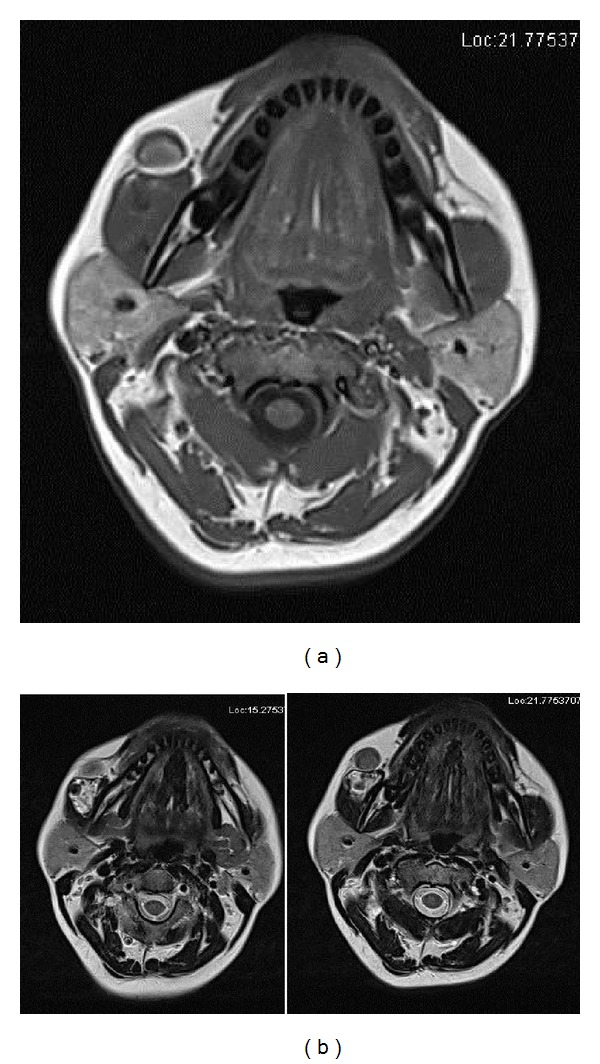
(a) Magnetic resonance imaging discloses isointense mass on T1-weighted MR image. (b) Isointense mass on T2-weighted MRI.

MRI revealed a well-defined isointense lesion adjacent to the anterior aspect of the right master muscle, appearing isointense on T2 and T1 sequences; also, hypointense foci with fatty infiltrations involving right masseter muscle were noted (Figures 8(a) and 8(b)).

FNAC yielded a thick brown material containing sheets of hemosiderin laden macrophages, many spindle cells, and degenerated erythrocytes.

Excisional biopsy was performed under general anesthesia employing stringent aseptic conditions and endotracheal intubation. Considering the location of the lesion, that is, the muscular plane in relation to masseter muscle, an extra oral submandibular approach was chosen to perform the biopsy.

**Figure 9:**
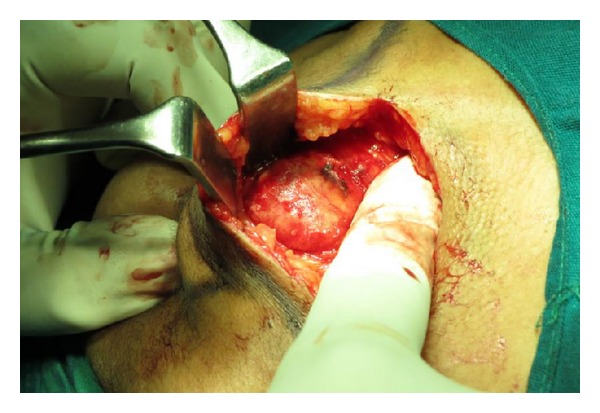
Extraoral approach revealed a well-defined mass adherent to the masseter.

Right submandibular incision measuring about 6 cm in length was marked to gain access to the masseteric area. Local anesthesia with adrenaline was infiltrated at the incision site. After the initial skin incision, parotidomasseteric fascia was then incised to gain access to the lesion within masseter muscle; care was taken not to involve any branches of facial nerve and blunt dissection was used. Once the masseter muscle was accessed, it was explored using blunt dissection and the masses of the lesion were localized. The lesion was found to have feeding blood vessels which were ligated before excision. The surgical site was checked for any residual bleeding keeping in mind the suspected nature of lesion. Upon achieving hemostasis, the surgical site was irrigated with povidone iodine solution and saline; suturing was done in layers using 3-0 vicryl and 4-0 prolene sutures (Figure 9).

**Figure 10:**
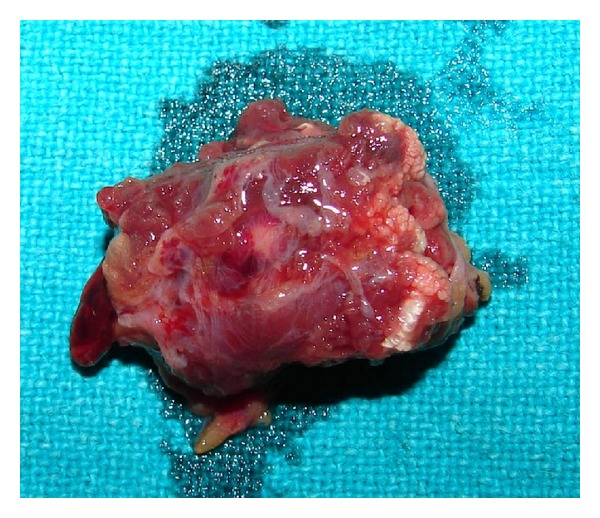
Gross specimen appeared purplish red with a feeder vessel.

The excised lesion grossly 3 × 3 cm in size was reddish blue in colour and firm in consistency, with small feeding blood vessels (Figure 10).

**Figure 11:**
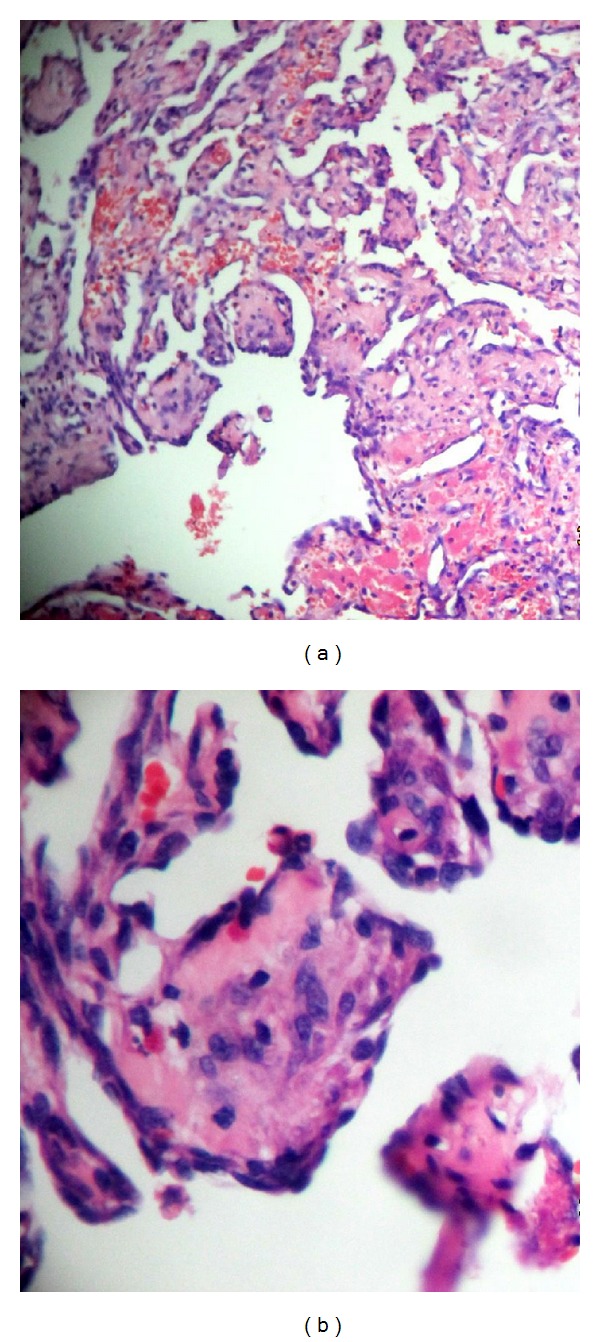
(a) Low power view of intravascular papillary processes within the lumen of dilated vein (H&E, original magnification ×40). (b) High power view showing papillary processes lined by benign endothelial cells (H&E, original magnification ×100).

Histopathological examination revealed a dilated vein with lumen showing papillary processes and anastomosing channels lined by benign endothelial cells. Fibrin deposits and areas of hemorrhage were also seen. No evidence of increased mitotic activity was observed and no atypia of the endothelial lining was evident (Figures 11(a) and 11(b)).

A final diagnosis of intravascular papillary endothelial hyperplasia/Masson's tumor was made.

## 3. Discussion

In 1922, Ewing described a rare and obscure intravascular endothelioma in the corpus cavernosum. He also described some cases of encapsulated, slow growing, subcutaneous tumors with abundant endothelial cells occurring in dilated varicose veins with previously obstructed circulation. These tumors grew in broad papillary masses formed by enlarged cells with hyperchromatic nuclei. In 1923, Pierre Masson first described an intravascular papillary proliferation formed within the lumen of inflamed hemorrhoidal plexus in a man which he believed was a neoplastic lesion and which he called Hémangioendothéliome végétant intravasculaire. Henschen described similar changes in nasal and laryngeal vessels which he considered to be a reactive phenomenon [4, 5].

Various terminologies have been put forth by different authors for this lesion including papillary fibroendothelioma and intravascular endothelioma, papillary proliferation of the endothelium, papillary endothelioma, Hémangioendothéliome végétant intravasculaire, L'endovasculite proliférante trombopoiétique, intravenous atypical vascular proliferation, intravascular angiomatosis, intravascular papillary endothelial hyperplasia, Masson's vegetant intravascular hemangioendothelioma, Masson's pseudoangiosarcoma, intravascular endothelial hyperplasia, Masson's lesion, and papillary endothelial hyperplasia [4]. The most accepted term being intravascular papillary endothelial hyperplasia (IPEH) was first used by Clearkin and Enzinger as quoted by Johraku et al. [6].

The pathogenesis of IPEH is poorly understood. One possible mechanism is a benign neoplastic process involving endothelial cell proliferation and papillary formation in the vascular lumen that undergoes degeneration and necrosis in the manner of a red infract. Alternative mechanisms include a benign endothelial proliferation arising from a thrombus as a variant of angiolymphoid hyperplasia with eosinophilia, a reactive process of endothelial cells induced by blood stasis and perivascular inflammation, and a pseudotumoral lesion caused by endothelial proliferation with papillary formation proceeded by an accumulation of thrombotic material, which serves to facilitate development of the lesion [3].

Various putative factors have been suggested preceding the appearance of the lesion including minor trauma [7]; Pins et al. suggested oestrogenic hormonal influence, and deranged paracrine signaling or inappropriate cytokine like fibroblast growth factor stimulation as quoted by Liu et al. [8].

Inoue et al. cited classification of IPEH by Hashimoto et al. as [1].

Type I is also called the primary or pure form which occurs within dilated vascular spaces. It is the most common subtype [7].

Type II is a secondary or mixed form that occurs in preexisting varices, cavernous or capillary hemangiomas, pyogenic granuloma, lymphangiomas, arteriovenous malformation, and blue rubber bleb nevus.

Type III or the undermined type is found in an extravascular location, the lesion develops in the bed of a hematoma and trauma is usually a prerequisite. It is the least common variant.

The IPEH in our case was a pure or Type I variant, as it was found to arise from a dilated vein.

Clinically, IPEH presents as a firm, tender, two-to-four cm nodule or mass with slight elevation and slow growth. It is usually characterized by red or blue discoloration of the overlying mucosa. This tumor most often occurs in the head and neck region (23%), lower extremities (17%), and fingers (16%) [8]; the rare sites affected include thyroid, orbit, parotid gland, masseter muscle, nose, mandible, pharynx, paranasal sinuses, and central nervous system [9]. Intraorally the common sites of occurrence are the lower lip, tongue, buccal mucosa, upper lip, mandibular vestibule, and angle of the mouth [3]. A seemingly rare site, the masseter, was involved in this case.

The lesion has been found to have a female preponderance, as in our case, with a male/female ratio of 1 : 1.3 and based on this finding a possible hormonal role has been suggested. The lesion commonly occurs in the middle age usually in the 5th decade with a mean age at onset being 42.6 years which was coinciding with the age of our patient [7].

Clinically, the lesion has been mistaken for and should be differentiated from mucocele, hemangioma, lymphangioma, angiosarcoma, hematoma, Kaposi sarcoma, hemangioendothelioma, thrombosed vein, phlebectasia, traumatic fibroma, melanoma, fibroepithelial polyp, nonodontogenic soft tissue infection, intramassetric abscess, cysticercosis, benign neoplasms of smooth muscle origin, and reactive and neoplastic neural lesions like traumatic neuroma, neurofibroma, and neurilemmoma [7, 10, 11].

IPEH can have varied appearance on imaging studies depending on the degree of thrombosis. In our patient, concentric calicifications were noted initially on conventional and digital radiographs. Performing an ultrasonographic study elucidated a well-defined cyst-like lesion located within the masseter. Color Doppler sonography revealed that the lesion lacked any obvious vascularity. Advanced imaging like CT and MRI enabled us to further identify the exact plane in which the lesion and associated calcific foci were located. The Hounsfield value of 100 HU and the isointense appearance on T2-weighted MRI indicated that the lesion was solid and not cystic. The lesion was also shown to have continuation with the right masseter muscle on CT and fatty infiltration of masseter on MRI which explains the difficulty in resecting the tumor mass alone.

Studies have shown that some IPEH masses are homogenous on CT and MRI, whereas others are nonhomogenous. MRI characteristics, such as hypointensity on T1-weighted images, hyperintensity on T2-weighted images, and some degree of contrast enhancement, are nonspecific to IPEH [12].

Such signals are probably related to the fact that lesions are likely to have varying amounts of both solid “parenchymal” tissue areas (papillary structures and thrombi) and anastomotic, stagnant, or low-flow vascular channels.

Areas of microcalcification have been noted in IPEH lesions. The presence of calcification is said to be typical in IPEH and the involved vessels may either be occluded or patent [13]. Phleboliths, however, are also common in patients with hemangiomas. Likewise, calcification can occur in hematomas and tumors, and they can follow necrosis of soft tissues. Moreover, on angiography, IPEH can manifest as either a vascular or avascular mass. MRI is usually superior as the initial diagnostic test for vascular malformations, and further investigations are usually not necessary in low-flow lesions. The CT, MRI, and angiographic patterns of IPEH can all simulate other benign (e.g., hemangioma) and malignant (e.g., angiosarcoma) conditions.

When IPEH causes bone erosion as a result of pressure effects, its radiologic features can be even more suggestive of malignancy. Therefore, while radiologic investigations can assess the nature of the lesion and contribute to management, they are not sufficiently characteristic by themselves to make a diagnosis of IPEH [14].

Thus, imaging plays considerable role in the diagnosis of IPEH to determine the extent of the lesion or its vascularity and guide the surgical management.

Histopathologically, in cases of IPEH associated with thrombi, an organizing thrombus is observed in an expanded blood vessel. The endothelial cells proliferated in a papillary pattern towards the lumen of the enlarged blood vessel from the area of the organizing thrombus. The structure of papillary proliferation was covered with no more than 2 layers of endothelial cells, and no atypia or mitotic activity was seen around the cores of fibrous connective tissue, which were frequently hyalinized and hypocellular [1]. Histological differential diagnosis of IPEH includes angiosarcoma, hemangioma, mucocele, intravenous, pyogenic granuloma, Kaposi sarcoma, spindle cell hemangioendothelioma, malignant endovascular papillary angioendothelioma or Dabska's tumor, and intravascular endothelioma [7].

Salyer and Salyer as referred by Robertson and Hernández, described the features which distinguish this lesion from angiosarcoma. (1) The lesions are well circumscribed and have a predominately intra- vascular location. (2) Extravascular lesions are associated with hematomas. (3) Rare but normal mitotic figures occur. (4) There is a lack of cellular atypia. (5) There are no necrotic areas. (6) Cells do not invade the perivascular space. (7) Solid areas exist with or without vascular differentiation. (8) Papillary fronds are one or two cells thick. (9) Papillary structures are mainly supported by a core of thrombotic material [4].

Constantino et al. as alluded by Robertson and Hernández hypothesized the stages through which the lesion progresses into 4 stages: the process probably starts with a recently formed thrombus and early endothelial proliferation occurs. Later, papillary projections develop when endothelial cells cover the irregular surface of the thrombus. In the more advanced stage, thrombotic material is slowly resorbed and papillary projections become smaller. At an end stage, the thrombotic material is completely resorbed and only a nodule composed of endothelial cells remains [4].

Ki-67 (MIB-1) is a large nuclear protein preferentially expressed during all active phases of the cell cycle but absent in resting cells. Avellino et al. have demonstrated the presence of a small number of Ki-67 (MIB-1) positive cells in IPEH tumors by immunohistochemistry, suggesting that these are slow-growing benign neoplasms and not reactive growths [3].

Immunohistochemically, IPEH reacts with vimentin, *α*-SMA, factor VIII, XIIIa, CD 31, CD 34, and ULEX europaeus agglutinin (UEA-1) corresponding to the endothelial cells being the proliferative component and the lesion having mesenchymal origin [1–4, 7]. CD 105 staining can help differentiate IPEH from angiosarcoma since this molecule is overexpressed only in angiosarcoma associated endothelial cells [3].

Sarode et al. cited various treatment modalities, most cases are cured by simple total excision with healthy margins. Cohen et al. used sclerotherapy (intralesional injection of a sclerosing agent “sodium tetradecyl sulfate”, causing compression and fibrosis of the blood vessels) followed by surgery with good esthetic results and minimal intra-operative bleeding. Endoscopic surgery has been used to treat an extensive IPEH of the sinonasal cavity. Noninvasively IPEH has been successfully treated by the beta-adrenergic antagonist nebivolol [15].

The prognosis of IPEH is excellent. Follow-up of some large series showed no evidence of local invasion or metastasis. Nevertheless, IPEH may recur if it arises in a primary vascular lesion which may itself recur or if the lesion is incompletely excised. Therapy in these cases should be planned according to the nature of underlying lesions. It has also been suggested that, in recurrent cases exhibiting strong immunolabeling of proliferative markers, the possibility of angiosarcoma should be investigated [15].

In our case, surgical excision with clear margins was performed and there was no evidence of recurrence at 6-month follow-up.

## 4. Conclusion

Intravascular papillary endothelial hyperplasia is a rare reactive lesion occurring notably in the head and neck region and can mimic many pathological lesions, both benign and malignant. Utilizing appropriate imaging modalities helps ascertain the location, proximity to vital structures, and the vascularity of the lesion aiding in diagnosis and surgical treatment planning, the gold standard for diagnosis being histopathological examination. It is vital to precisely diagnose this lesion so that it is neither inadequately nor aggressively managed both of which are crucial to prevent recurrence and morbidity.
